# NAA60 (HAT4): the newly discovered bi-functional Golgi member of the acetyltransferase family

**DOI:** 10.1186/s13148-022-01402-8

**Published:** 2022-12-21

**Authors:** Federica Donnarumma, Valeria Tucci, Concetta Ambrosino, Lucia Altucci, Vincenzo Carafa

**Affiliations:** 1 Biogem, Molecular Biology and Genetics Research Institute, Ariano Irpino, Italy; 2 Department of Precision Medicine, University of Campania “Luigi Vanvitelli”, Vico De Crecchio7, 80138 Naples, Italy; 3 Department of Science and Technology, University of Sannio, Benevento, Italy

**Keywords:** Epigenetics, Protein post-translational modification, Protein acetylation, Histone acetyltransferases, NAT, NAA60, HAT4, NatF, NAT15, Golgi, Cancer

## Abstract

Chromatin structural organization, gene expression and proteostasis are intricately regulated in a wide range of biological processes, both physiological and pathological. Protein acetylation, a major post-translational modification, is tightly involved in interconnected biological networks, modulating the activation of gene transcription and protein action in cells. A very large number of studies describe the pivotal role of the so-called acetylome (accounting for more than 80% of the human proteome) in orchestrating different pathways in response to stimuli and triggering severe diseases, including cancer. NAA60/NatF (N-terminal acetyltransferase F), also named HAT4 (histone acetyltransferase type B protein 4), is a newly discovered acetyltransferase in humans modifying N-termini of transmembrane proteins starting with M–K/M-A/M-V/M-M residues and is also thought to modify lysine residues of histone H4. Because of its enzymatic features and unusual cell localization on the Golgi membrane, NAA60 is an intriguing acetyltransferase that warrants biochemical and clinical investigation. Although it is still poorly studied, this review summarizes current findings concerning the structural hallmarks and biological role of this novel targetable epigenetic enzyme.

## Background

**Fig. 1:**
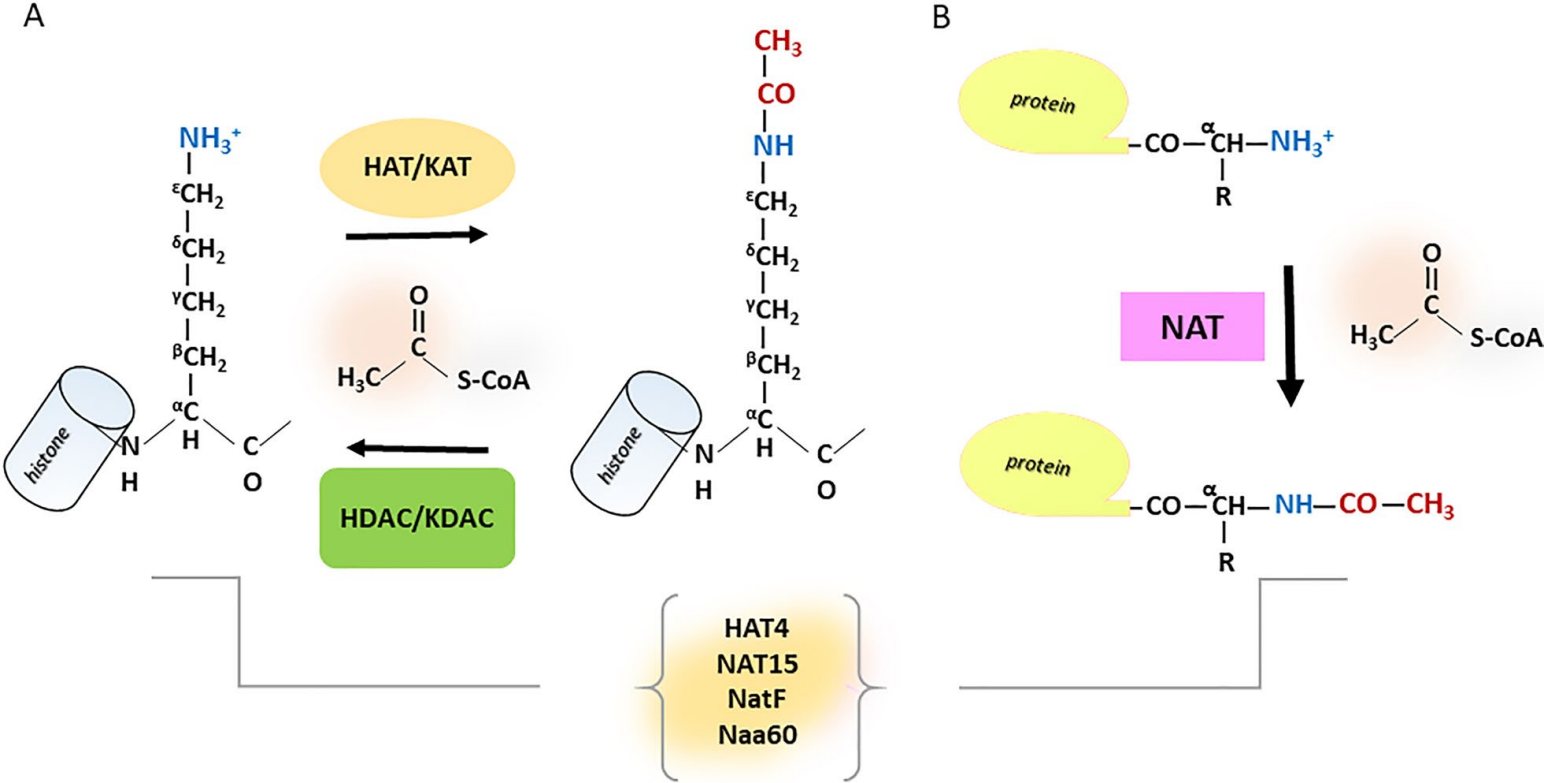
HAT/KAT and NAT enzymatic action. The NAA60/NatF bi-functional behaviour: (**A**) HAT/KAT enzymatic activity; (**B**) NAT enzymatic activity

The post-translational attachment of functional groups to polypeptide chains enables cells to specifically and rapidly react to numerous endogenous and exogenous stimuli [1, 2]. Modification of proteins at their N-termini is a widespread phenomenon highly conserved across evolution from viruses to higher eukaryotes. Protein acetylation, one of the most studied post-translational modifications (PTMs) along with phosphorylation, methylation, SUMOylation and ubiquitination, is a major chemical modification where an acetyl group from acetyl coenzyme A (Ac-CoA) is transferred to a specific amino acid residue [2]. In humans, around 90% of the whole proteome is co-translationally acetylated at the Nα-termini of nascent polypeptides. Acetyl, pyruvoyl, formyl, glucuronyl, α-aminoacyl, α-ketobutyryl, pyroglutamyl, murein, glucose [3, 4] and carbon dioxide [5] are known as acylating groups. The target most susceptible to protein acetylation is the lateral chain of lysines on their ε-amino group. Since acetylated lysines were initially found in histones, the first members of the acetyltransferase enzyme family were called histone acetyltransferases (HATs) [1] (Fig. 1A). However, lysine acetylation is not restricted only to histones, and the enzymes were subsequently renamed lysine (K) acetyltransferases (KATs) [6] to better distinguish them from the HATs group. The acetylation reaction on N-termini is catalysed by N-terminal acetyltransferases (NATs) on different starting amino acid substrates [7] (Fig. 1B). Unlike N-terminal acetylation, which is irreversible, lysine acetylation is reversible and tightly controlled. In support of this finding, several families of lysine deacetylases (KDACs) counteract the action of KATs [8, 9]. KATs and KDACs are well poised to modulate dozens of cell functions in a wide range of conditions. This behaviour also introduces the concept of *reader* proteins in addition to the *writers* (HATs) and *erasers* (histone deacetylases HDACs) [1]. To date, 18 deacetylase enzymes grouped into four classes have been characterized in humans: 11 HDACs and 7 sirtuins. Upon the depletion of acetyl groups, these enzymes maintain a finely tuned equilibrium of lysine acetylation in histone and non-histone proteins [10, 11]. HDACs (HDAC1–11) belong to Zn^2+^-dependent class I, II and IV [12], whilst class III enzymes (SIRT1–7) have a NAD^+^-dependent activity [13].

Compromised acetylation can trigger severe pathologies such as tumours, neurodegenerative syndromes and cardiovascular disorders [14]. However, the biological outcomes linked to acetylation, as well as to the synergism and cross-regulation of the acetylome itself, are still unclear. As clinical research is becoming increasingly focused on developing personalized pharmacological therapies for serious diseases, the interest in acetyltransferase biology is steadily growing. Here, we describe the Golgi NAA60/NatF (N-terminal acetyltransferase F), also named HAT4 (histone acetyltransferase type B protein 4). It is the newly investigated NAT member of the big acetyltransferase family and, undoubtedly, represents a worthy protein model to better unveil the relationship between protein acetylation and epigenetics in the field of physiopathology and nanomedicine.

### Acetyltransferase enzymes: an overview

A general overview of the most characterized acetyltransferases is provided in Table 1 [15]. Enzymes that catalyse the acetylation of histones are called HATs (Fig. 1A). HATs can acetylate both nucleosomal and free histones. Type A HATs (A-HATs) mainly exert their enzymatic action at a nuclear level on the histone octamer. They are classified into three groups: 1) the general control nonderepressible 5 (GCN5)-related N-acetyltransferase (GNAT) family, including KAT2A and KAT2B; 2) the MYST family, the largest group, consisting of KAT5/6A/6B/7/8; and 3) the p300/CBP family, comprising KAT3A and KAT3B [16].

**Table 1:** Major eukaryotic acetyltransferases

	Family	Name	Alternative name	Cell localization	Amino acid target
HAT	GNAT	HAT1	KAT1	Nucleus	K
		**NAA60**	NatF/HAT4/NAT15	Golgi	K
		KAT2A	GCN5	Cytosol	K
		KAT2B	PCAF	Nucleus	K
NAT		NatA	*	Cytosol	A/S/T/G/V/C
		NatB	*	Cytosol	MD/ME/MN/MQ
		NatC	*	Cytosol	ML/MI/MF/MY/MK
		NatD	*	Cytosol	SGRGK
		NatE	*	Cytosol	M-S/T/A/V/L/I/F/Y/K
		**NatF**	NAA60/NAT15/HAT4	Golgi	M–K/M-A/M-V/M-MS
		NatG	NAA70	Chloroplast	M/A/S/T
		NatH	NAA80	Cytosol	DDD/EEE
HAT	MYST	KAT5	Tip60	Nucleus	K
		KAT6A	MOZ/MYST3	Nucleus	K
		KAT6B	MORF/MYST4	Nucleus	K
		KAT7	HBO1/MYST2	Nucleus	K
		KAT8	MOF/MYST1	Nucleus	K
HAT	p300/CBP	KAT3A	CBP	Nucleus	K
		KAT3B	p300	Nucleus	K

HAT and NAT families’ members are grouped. The alternative names, the cell localization and the acetylated peptide target are reported

Type B HATs (B-HATs) are cytoplasmic enzymes and acetylate-free histones, thus impacting on the structure of the nucleosome at protein and DNA level [17]. B-HATs differ from A-HATs, which act on histones already assembled into chromatin fibres [18]. Only a small number of B-HATs have been investigated: HAT1, for its acetylation of H4 on lysines 5/12 with HAT2 as a cofactor [19, 20]; Rtt109, for its targeting of H3 on lysines 9/27/56 [21]; and HatB3.1, for its acetylation on H3 [22]. HAT1 is the only eukaryotic B-HAT widely expressed across the phylogeny and is conventionally considered a paradigm for B-HATs [23]. Structurally, HAT1 belongs to the GNAT family and, functionally, it has a pivotal role in chromatin organization [20, 23].

Here, we focus on NAA60, one of the recently identified members of the B-HAT group, and it shares some features showed by the archetype B-HAT HAT1.

Unearthed more than 50 years ago, N-terminal acetylation is one of the most common protein modifications, whose reaction is catalysed by different NATs. NATs move acetyl groups from Ac-CoA molecules to the positive free α-amino tail of the newborn polypeptide extremity (Fig. 1). The added acetyl groups neutralize the exposed positively charged target, robustly impacting the electrostatic attributes of the protein and blocking the nascent N-segment from further chemical modifications [2, 24]. The acetyl group is usually co-translationally attached to the starting methionine of the nascent amino acid chain or after its release by ribosomal methionine aminopeptidase actions [25]. Not every conceivable NAT protein substrate can be utterly N-terminal acetylated, and they can sometimes simultaneously exist in an unacetylated and acetylated state. NATs actions bestow a role in regulating a wide range of molecular events, such as protein–protein interactions [26], protein subcellular distribution [27, 28], protein folding [29], protein delivery, protein half-life and degradation signalling [30]. To date, six human NATs have been discovered and characterized [2, 31]. NatA, NatB and NatC are oligomer enzymes made up of two or more assembled catalytic protomers and are involved in around 80% of N-terminal acetylation reactions in humans [7]. NatD (NAA40) is monomeric and reported to act independently [32, 33]. NatE (NAA50) acts as both a monomer and a heterodimer with NatA [34]. The last NAT member found in animals is NatH, also named NAA80 [35–37].

Each NAT mainly acts on the first two residues of the N-terminal tail of specific peptide substrates. The NatA catalytic subunit NAA10 can switch its substrate specificity following the binding affinity of its accessory counterpart NAA15 [38]. The heterodimer NAA10/NAA15 undergoes conformational changes that alter the catalytic site, enabling the acetylation of amino termini in which, upon excision of the initiator methionine (M1) by aminopeptidases, the adjacent amino acids are more exposed on the new N-segment. In contrast, free NAA10 is reported to primarily acetylate the acidic starting residues glutamate or aspartate [39]. NAA20 also shows substrate specificity for proteins with M1 flanked by an acidic amino acid. NAA40 specifically transfers an acetyl group on the starting N-terminal serine of histones H2A and H4, whilst NAA30 and NAA50 modify unprocessed N-terminal methionines followed by either a basic or hydrophobic residue in the second position [7]. As regards NAA80, preferentially displays preferences towards acid sequences starting with DDD and EEE in vivo [35, 37] whilst in vitro, acts on MDEL and MDDD starting polypeptides [36].

Although they exert different actions, HATs and NATs share some structural features. NATs belong to the GNAT superfamily, which also includes a few members of the KAT subfamily. All the known NATs are distributed in the cytosol and/or associated with ribosomes. Interestingly, NatF (NAA60) is anchored to organelle membranes in human [40] whilst has been recently discovered that it localizes to the plasma membrane in plants [41]. Another unusual organelle NAT, NatG (NAA70), was recently found in chloroplasts of *Arabidopsis thaliana* and acetylates proteins starting with M, A, S and T [42].

### Epigenetic role of acetyltransferases

As described in the previous sections, NAA60 displays features shared by several acetyltransferase family members, including NATs and HATs that are endowed with key epigenetic properties involved in regulating gene expression. HATs have various substrate specificities for both histone and non-histone proteins [43] and are therefore involved in numerous pathways. HAT1 promotes histone acetylation of H2A at lysine 5 and di-acetylation of H4 at lysines 5 and 12 and is thus an important regulator of chromatin organization and gene expression [10].

The MYST family member KAT5, also known as Tip60, is part of the multisubunit nucleosomal acetyltransferase of H4 (NuA4) complex responsible for acetylation of histone H4 [44], and is recruited by many transcription factors on their target promoters, thereby activating transcription [45]. Due to this activity, Tip60 is associated with DNA damage responses through the transcription factor p53; however, it also has transcription-independent roles through its binding with ATM and DNA-PKcs and is involved in their activation in response to double-stranded DNA break stimuli [45]. KAT8, also known as MYST1 or MOF, exploits the main acetylation function forming the multisubunit male-specific lethal (MSL) complex involved in histone H4 acetylation at lysine 16, the major H4 modification. Additionally, it is part of the human non-specific lethal (NSL) complex, which acetylates histone H4 at lysines 5/8 [46]. MOF also acetylates non-histone proteins, including transcription factors such as p53, whose activity is important in driving cells towards the apoptotic cascade upon induction of *Bax* and *Puma* genes [47]. KAT8 also promotes the activation of EGFR signalling [48] and is a regulator of the autophagy machinery regulated by ATG7 and BECLIN1 [49].

KAT7 (MYST2) preserves chromatin organization at centromeres. Specifically, KAT7 antagonizes the invasion of pericentric heterochromatin into flanking centromeric chromatin by promoting the removal of trimethylated H3 on lysine 9, thereby allowing CENP-A deposition and interaction with M18BP1 protein, and maintaining chromosomal architecture during chromatid separation [50]. KAT6A (MYST3) inhibits cell senescence through the INK4a-ARF pathway and is necessary to maintain normal acetylation levels of H3K9 and H3K27 at the transcriptional start sequences of *Cdc6*, *Ezh2*, *E2f2* and *Melk* [51]. KAT6B (MYST4) regulates the MAPK signalling pathway upon H3 acetylation [52].

KAT2A (GCN5), a member of the GNAT family, is involved in a wide range of biological functions such as gene regulation, cell proliferation, metabolism and inflammation [53]. KAT2A has a wide impact on Ac-CoA metabolism and on the acetylation of protein transcriptional coactivators, such as PGC-1α, which orchestrates the expression of genes involved in energy metabolism and mitochondrial biology [54].

The two proteins KAT3B (p300) and KAT3A (CBP) comprising the p300/CBP family are often considered to work as a single system due to their structural and functional similarities. They exert numerous actions, serving as a bridge between DNA binding sites and general transcription factors, allowing chromatin relaxation through their HAT activity, working as coactivators for nuclear receptors, and acetylating transcription factors such as c-Myb, c-Myc, CREB, c-Fos, c-Jun, STAT, BRCA1, FOXO 1/3a/4, SMA/MAD, HIF-1α, E2F and RUNX [55]. Like HATs, NATs also have important roles in regulating cellular functions. The human NatC complex acetylates a wide range of proteins, and loss of the NatC catalytic subunit affects mitochondrial structure and function [56]. Despite its name referring to N-acetylase activity on proteins, NAT10 is mainly involved in the formation of N^4^ -acetylcytidine modification in 18 S rRNA [57], mRNA [58] and tRNA [59]. For example, it acetylates CEP170 mRNA, enhancing its translation efficiency [60]. In addition to this role, Nat10 also acetylates proteins such as p53 activating it [61]. Particularly, NAT10 activated p53 through K120 acetylation, opposing the action of Mdm2 and mediating its degradation thanks to its E3 ligase activity. When DNA damage occurs, NAT10 translocates into the nucleus and activates p53 downstream cascades, thus modulating the cell cycle and apoptosis [61]. In yeast, NatA is involved in adaptation control systems: mutations in *NatA* are followed by an alteration in the transposon system, in sub-telomeric genes and in genes responsible for pheromone response and encoding for mitochondrial and ribosomal proteins [62]. NatA complex contains NAA10 and NAA15 subunits and acetylates about 40% of the human proteome [63]. NatA interacts with the Huntingtin (Htt) yeast two-hybrid protein K (HYPK) protein [64]. The NatA / HYPK complex has a dual role from which both proteins benefit: it prevents the Htt aggregation and strengthens the N-acetylase activity of NatA [65]. NatB predominantly regulates protein folding, and when it is mutated protein aggregation occurs; this event is recognized as a sign of cellular stress [66]. NatD downregulates transcriptional expression levels of Slug, a master regulator of epithelial–mesenchymal transition [67]. NatE activity is mediated via NAA50 or in complex with NAA10/NAA15 with HYPK [64, 65].

Given the major impact of acetyltransferases and the modifications they promote at the epigenetic level, the newly discovered member of HAT and NAT families, NAA60, is a fascinating enzyme that warrants further study as a new epigenetic marker in both physiological and pathological conditions.

### Acetyltransferases in diseases

Compromised acetylation patterns can result in altered phenotypes and insurgence of diseases (Table 2). Altered expression of the *MYST1* gene is found in some primary cancers [46]. For example, *MYST1* is upregulated in non-small-cell lung cancer (NSCLC) tissues and is associated with large tumour size, advanced disease stage, metastasis and poor prognosis. MOF acetylates Nrf2 at lysine 588, increasing its nuclear retention and transcription. Importantly, MOF is crucial for anti-oxidative and anti-drug responses and influences tumour growth and chemoresistance in an Nrf2 acetylation-dependent manner. MOF may therefore represent a therapeutic target for the treatment of NSCLC [68]. MYST1 expression is increased in glioblastoma and promotes tumour progression by activating EGFR signalling [48]. *MYST1* downregulation inhibits cell proliferation, inducing cell cycle blockage in G2/M phase, with a consequent reduction in levels of cyclins, EGFR, CDK, Erk1/2 and AKT, and increased expression of p21/Waf [48]. MOF is also involved in kidney cancer and could be considered a marker for cancer diagnosis [46]. In fibrotic diseases, such as systemic sclerosis, overexpression of MYST1 in tissues abrogates TGFβ-mediated effects on the autophagy process through BECLIN1 and ATG7 proteins [49].

**Table 2:** Acetyltransferases involvement in disease outcomes

Name	Target	Disease	References
MYST1	NFR2	Non-small-cell lung cancer	[68]
	EGFR	Glioblastoma	[48]
	TGFβ	Fibrosis	[49]
Tip60	Neural plasticity genes	Neuronal disease	[69]
	DNA repair genes	Breast cancer	[70]
MYST2	β-catenin	Alzheimer’s disease	[71]
	MLLX	Acute myeloid leukaemia	[72]
MYST3	KAT6a/MYST3	Syndromic developmental delay	[73]
MYST4	MAPK	Noonan syndrome-like phenotype	[52]
	MYST4 OE	High-grade serous carcinoma	[75]
GCN5	GCN5 OE	Hepatocellular carcinoma	[76]
	Egr-1, E2F1, Bim	Brain injuries	[76]
p300/CBP	cAMP-PKA, p53, ER	Lung cancer	[123]
NAT10	CEP170	Multiple myeloma	[60]
NatA	β-catenin /cyclin D1, HIf1a, TSC2/mTOR, E-cadherin, p21, PIX/Cdc42/Rac1, MLCK, DNMT1	Cancer	[79]
NatB	Tropomyosin, CDK2, Hippo/YAP, ERK1/2	Hepatocellular carcinoma	[84]
NatD	Slug	Lung cancer	[67]
NatF	INFα	Influenza A virus	[103]

The acetylated targets and the diseases related to compromised acetylation are reported

Alterations in the acetylation state at the neural level are involved in many neurological diseases. The early silencing of Tip60 neuroplasticity genes affects synapse morphology, locomotion and short-term memory. Conversely, increasing Tip60 levels prevents locomotion and short-term memory deficits [69]. Tip60 also acetylates p53 on K120, modulating its role as a tumour suppressor, although it is downregulated in several breast cancers carrying a *p53* mutation. This means that when Tip60 is downregulated, it cooperates in tumour suppression independently of its acetylase activity on p53. Low Tip60 levels are reported to lead to DNA repair defects, prompting tumorigenesis [70].

KAT7 (MYST2) has effects on β-catenin or the Wnt inhibitor Dkk1 in Alzheimer's disease positively regulating tau phosphorylation and exacerbating cognitive deficits [71]. Low expression of KAT7 (MYST2) rapidly determines a lack of H3K14 and H4K12 acetylation, slowing cell growth and inducing apoptosis and differentiation of leukaemia cells. Acetylated histones likely act as adapters for the binding of proteins such as BRD4 and AF4 on gene promoters that are important for myeloid cell proliferation, including *PBX3*, *SENP6* and *MEIS1* [72].

Mutations in *KAT6A* (*MYST3*) were recently identified as a cause of syndromic developmental delay [73]; truncating mutations in the transactivation domain of KAT6A are causative of intellectual disability syndromes [74] and gastrointestinal complications [73].

Insufficiency of *MYST4* gene is mapped in patients with Noonan syndrome, where H3 acetylation is affected, suggesting its impact on neural, craniofacial and skeletal morphogenesis through regulation of the MAPK signalling pathway [52]. MYST4 is also strongly expressed in ovarian high-grade serous carcinomas (HGSCs) and is prognostic for reduced patient survival at an advanced stage [75]. A similar picture is found in hepatocellular carcinomas (HCCs) due to more aggressive tumour progression [75]. GCN5 is overexpressed in more than 50% of human HCCs [76]. Downregulation of *GCN5* inhibits HCC cell proliferation and xenograft tumour formation and reduces protein levels of the proliferation markers PCNA and AIB1, whilst increasing protein levels of the cell cycle inhibitor p21^Cip1/Waf1^ [76]. Inactivation of GCN5 in cultured cerebellar granule neurons leads to apoptosis activation. Furthermore, the loss of GCN5 activity and induction of E2F1, Bim and Egr-1-related pathways results in premature brain injuries and haemorrhage in rats when cell acetylation homeostasis is impaired [76]. As for the p300/CBP family, deregulation in *CBP* gene expression was found in haematological malignancies [77] as well as in congenital malformation and mental retardation. *p300* gene is implicated in leukaemia, and mutations are observed in gastric and colorectal carcinomas [78].

Human NATs are also involved in acute diseases. Numerous NAT subunits are upregulated in different forms of cancers [79] and, as described for point mutations in *NAA10* gene, can be involved in the aetiology of intellectual disabilities and rare genetic diseases [80–82]. NATs act as both oncoproteins and tumour suppressors in human cancers. Alterations in NATs expression in vitro lead to cell cycle arrest, autophagy or apoptosis activation, highlighting that NATs target dozens of cellular processes. Specifically, NAA10 (the catalytic subunit of the NatA complex) is associated with many signalling molecules, including β-catenin/cyclin D1, HIF-1α, PIX/Cdc42/Rac1 and DNA methyltransferase1/E-cadherin. NAA10 acts as both a tumour suppressor and an oncogene [79]. NAT10 is strongly upregulated in multiple myeloma patients [60]. RNA and ribosome profiling sequencing identified CEP170 as an important downstream target of NAT10. CEP170 also promotes cell proliferation and chromosomal instability in myeloma. In vitro studies report that NatA acetylates the Huntingtin protein, promoting its aggregation [83].

Human NatB acetylates more than 10% of the proteome, and this modification is fundamental for proper actin structure and function in the cytoskeleton. Cytoskeleton changes are known to influence tumour progression by acting on motility, invasion, survival and proliferation of cancer cells, thus pointing to the cytoskeleton as a promising therapeutic target. NatB subunits are upregulated in over 50% of HCCs, and this pattern is associated with vascular invasion [84]. In addition, NatB depletion inhibits proliferation and tumour formation in HCC cells as well as impairing DNA replication and hampering cell cycle progression from S to G2/M phase due to a loss of acetylation on tropomyosin and CDK2, two NatB substrates. This has an effect on the actin network and tight junctions, suppressing the proliferative pathways ERK1/2 and Hippo/YAP [84]. Furthermore, NatB affects levels of the conserved pathway components MAPK, PP2AC and Grb2/Drk [85]. NatD promotes propensity towards migration and invasiveness in vitro and in vivo. Lack of NatD abrogates the epithelial–mesenchymal transition of lung cancer cells by directly repressing expression of the transcription factor Slug upon N-terminal acetylation of histone H4, which contrasts histone H4 phosphorylation on serine 1 [67].

Together, these findings indicate that a more comprehensive analysis of the acetylome could pave the way towards a better understanding of the onset of severe pathologies and that acetyltransferases are crucial epigenetic modulators of disease outcomes.

### A new HAT, a new NAT in humans

NAA60 is conserved among animals, and homologues might also be expressed across the plant kingdom, as recently found in *Arabidopsis thaliana* [41]. Proteome analysis revealed that this protein contains the so-called GNAT functional domain (55–156 aa), also present in HAT1, HAT2 and HatB3.1, and it was therefore initially named NatF [31]. Sequence alignments showed that human NAA60 shares more than 95% identity with the *Mus musculus* form and more than 50% with the Drosophila homologue [86].

NAA60, is one the newest and least studied human NAT. Differently from the highly conserved analogues NAA10/NAA50, NAA60 and NAA80 are not expressed in fungi. Unlike other mainly nuclear and cytoplasmic HATs and NATs, this unusual acetyltransferase is localized in the Golgi membrane [40, 87]. Depletion of NAA60 leads, in fact, to Golgi fragmentation [40]. Pioneering studies on human NAA60 revealed extensive redundancies in substrate preferences with NAA30 and NAA50 [31]. However, recent data also showed the significant presence of transmembrane proteins in the predicted NAA60 acetylome profile, suggesting that evolution conferred the ability to acetylate the cytosolic NH_2_^+^ extremities of membrane proteins as a result of its membrane sub-localization [40].

### Function and mode of action of NAA60

Proteome analysis coupled with multiple peptide sequence alignments allowed to assess that the newly identified NAA60 was also a NAT [31]. Mass spectrometry experiments carried out on the recombinant NAA60 incubated with a peptide library generated from natural proteomes helped to identify numerous N-acetylated peptides among the acetylated target of other NATs. Thus, following the NAT nomenclature system [88], the new protein was named NAA60 and its activity NatF, because of its preference for the protein N-terminal side. Remarkably, the preferred N-terminal branches included M–K, M-A, M-V and M-M peptide couples with yet uncharacterized target NATs. Interestingly, a further study revealed that several M–K starting polypeptides were acetylated in Homo and Drosophila, whilst the same modification was not identified in yeast, indicating the existence of more NAT-specific targets in higher organisms [86].

### Structure–function relationship hallmarks of NAA60

**Fig. 2:**
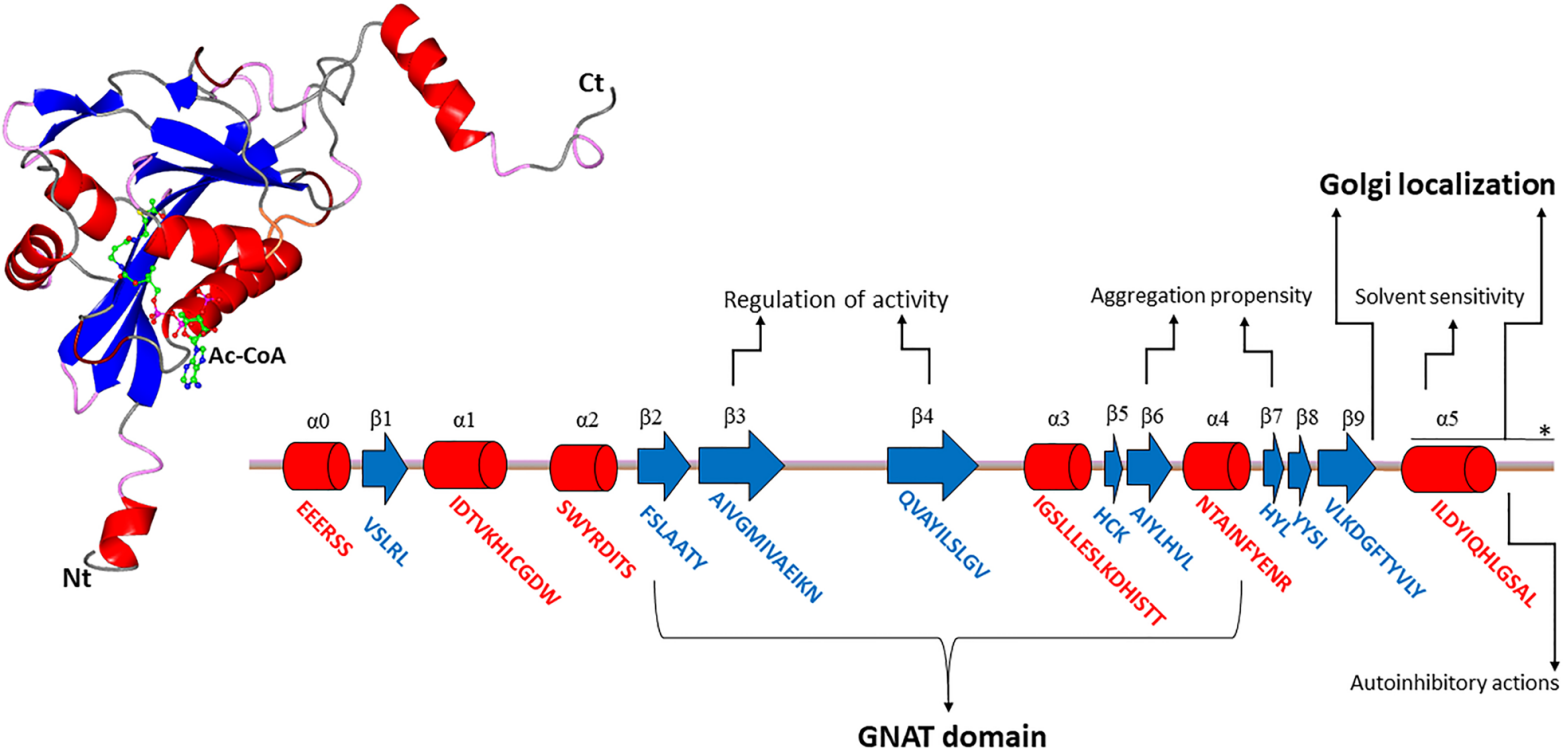
NAA60 structural–functional insights. The main structural hallmarks of tertiary and secondary structures are highlighted (red, α-helix; blue, β sheets; grey-pink, loops). The structure is referred to the crystalized form (1–212 residues) PDB code 5GHZ. With (*) is reported the missing α-helix not present in 5GHZ crystal (shorter than the 1–242 wild-type protein)

Meaningful structural and biochemical insights into NAA60 came from physicochemical studies performed on different recombinant variants and from comparison with other NATs. NAA60 comprises a central domain showing the typical GNAT folding, made up of a mixed α-β fold with a conserved Ac-CoA binding site, also shared by other known NATs. The total structure consists of nine β-sheets and six α-helices (Fig. 2). Loops are also important structural elements that give the protein a peculiar profile. Between β3 and β4, NAA60 has an extended 20-residue loop circumscribing a small subdomain with a well-structured chemical environment. Furthermore, the β7 and β8 strands are organized as a roughly antiparallel β-hairpin motif noticeably dissimilar to that of NAA50, although its 3D structure resembles that crystalized for NAA60 [89, 90]. The amino and carboxyl-terminal regions form helicoid secondary structure elements obtruding from the GNAT domain. Enzymatic studies revealed that the full-length protein (242 residues) is less active than the truncated variants, showing that the external region (residues 200–242) may exert some auto-inhibitory actions. Multiple alignment analyses revealed sequence homology with other NATs within the N-terminal 180 residues, but a sequence mismatch at the C-terminal domain, important for Golgi localization [40, 87, 89]. In the conserved NAT region, NAA60 exhibits the highest divergence in the β3-β4 (residues 73–94) and β6-β7 (residues 165–173) loops, which in humans are longer than other eukaryotic NATs. The loops at β-strands 3–4/6–7 adopt specific tertiary structures. The β3-β4 loop twists directing on the core peptide substrate domain; the loop between strands 6 and 7 holds a β-hairpin structure with two short β-segments bordering the substrate-binding site, in a paradigmatic way also shared by all other NATs [90]. Crystallographic data showed that the C-terminal branch emerging from the GNAT core folds as an amphipathic helix (α5) that interacts with a neighbour molecule via hydrophobic interactions between α5 and a hydrophobic furrow between the amine extremity of β1 and β3 strands of the proximal molecule. The C-terminal segment following this helix adopts a wrapping β-turn motif, which similarly touches the neighbour protein molecule via hydrophobic interactions. Residues 182–216 enclose a structural segment important for NAA60 localization on the Golgi apparatus. Specifically, the solvent-exposed amphipathic α5 (residues 190–202) contains a sequence rich in hydrophobic amino acids (I190, L191, I194, L197 and L201) located on one side and hydrophilic residues on the other. This fine-tuned charge distribution might allow the interactions between NAA60 and the Golgi membrane, as membrane proteins crossing the lipid bilayer [89]. The long 22-residues β3-β4 loop (residues 73–94) is absent in most NATs, including SpNAA10, NAA50 and NAA40, whose β-strands 3 and 4 are only connected by a small β-hairpin. In particular, the aspartic acid residues 81 and 83 in this loop mediate hydrogen bonds with histidines 138 and 157 of β-strands 5 and 7, respectively; the backbone nitrogen of isoleucine 77 interacts with phenol oxygen of tyrosine 136 via a water-mediated H bond. Further, E80, D81 and I84 make H bridges involving Y164, T176 and Y180 of the β6 strand. Several residues of the long β3-β4 loop (I77, I84 and L85) also interact with residues of the β5-β6-β7 core (V95, A134, Y136 and V178) by van der Waals forces. Interestingly, despite the very large number of contacts between the above-mentioned β-elements, the mutational analysis revealed that the conformation of this loop could be quickly disturbed. Specifically, substituting residues D81, I84, or Y164 in A or F results in altered catalytic parameters and has a mild effect on enzymatic efficiency. This suggests that, when locally changing some of the residues involved in these contacts, the structure of the whole enzyme might be globally perturbed, only slightly affecting substrate–peptide binding and catalysis. Indeed, point mutations in these regions impact on protein stability, which could trigger protein aggregation, as reported in other protein models [91]. In the presence of Ac-CoA and CoA-like molecules, an increase in T_m_ of the thermal denaturation curves around 10 °C was observed [90]. The β3-β4 loop, which is a short turn in other NATs, is involved in the regulation of NAA60 activity. This loop acts as a gate that partly wraps the substrate-binding region; this disordered section might be more sensitive to environmental physicochemical parameters, thus affecting protein stability at both local and global levels, as described in other protein models [92]. The solvent-exposing K79 may in fact have a putative role in shifting this gating loop under the surrounding solvent exposition. The negative residues E80, D81 and D83 interact with the positive residues H138, H159 and H158 to preserve the β3-β4 loop conformation, thus contributing to achieving substrate-binding specificity. Indeed, alanine mutants exhibit a twofold increase in NAA60 activity [90].

The superposition of NAA60 and NAA50 X-ray structures highlighted divergences in the β7-β8 hairpin region, although the GNAT domain was similar in sequence and global structure. In NAA60, the β7-β8 hairpin is near the α1-α2 loop, increasing greater compactness in the substrate-binding region than in Naa50, where this segment is more flexible. Depletion of the C-terminal region of NAA60 results in a conformational change that markedly modifies the geometricity of the substrate-binding region, which might influence the way in which the substrate interacts with the catalytic cavity. Interestingly, in the truncated NAA60 variant (residues 1–199) crystals, the misplaced β7-β8 hairpin favoured the substrate to access the active core in a second way that would effortlessly allow the accommodation of histone H4, thus potentially explaining the KAT propensity of NAA60 from a structural point of view [90]. Although the main activity remains the NAT activity, deeper conformational-functional data which could better clarify the KAT activity on H4 needs to be further assessed both in vitro and in vivo.

Focusing on the NAA60 enzymatic activity, residue F34 is involved in the positioning of the Co-A cofactor for acetyl transfer. F34 (equivalent to F27 in human NAA50) facilitates the binding of the N-terminal methionine of the peptide substrate via hydrophobic bonds. However, a hydrophilic malonate molecule is also found where the N protruding M should interact, suggesting that F34 may further allow the accommodation of hydrophilic substrates. In addition, the orientation of the F34 side chain might influence the positioning of the terminal side of the CoA close to the amine substrate. Indeed, the F34A mutant does not exhibit this behaviour. Thus, F34 plays a dual role both in peptide substrate–enzyme interactions (recognition) and in proper Ac-CoA positioning, modulating acetyl transfer activity (action).

A comparison of the active sites of NAA60 and NAA50 showed that the catalytic and substrate-binding key residues are highly conserved in both NATs. For catalysis, NAA60 employs residues Y97 and H138 (Y73 and H112 in the analogue NAA50). Biochemical studies on the derivatives Y97A, Y97F, H138A and H138F found that enzymatic action was abolished, indicating that the protein follows the same molecular mechanism involving Y97 and H138 as that observed in the NAA50 counterpart. Residues Y38, N143 and Y165 surround the malonate and interact through H bonds or water bridges. Although malonate is negatively charged, unlike the positive lysine ε-amine group, similar hydrophilic interactions occur when the amine substrate is in the same position, since Y38, N143 and Y165 are chargeless. Remarkably, the activity of Y38A, N143A and Y165A mutants was considerably decreased compared to that of the parent protein, underscoring the fact that these residues have a pivotal role in substrate binding [89].

### Aggregation propensity and functional forms of NAA60

Crystallographic studies on the NAA60/CoA complex revealed that the enzyme undergoes conformational changes upon binding peptide substrates, resulting in protein aggregation in vitro in a condition mimicking the physicochemical cell background. In the presence of its substrate, NAA60 crystallized as a dimer (PDB codes: 5ICV (monomer X-ray structure); 5ICW (dimer X-ray structure) [89]. The loop connecting β6 and β7 of the first monomer protrudes into the catalytic site of the close monomer, leaving no free space for another peptide substrate to accommodate it. Numerous contacts play a role in homodimerization: lysine 171 from each protomer branches out van der Waals contacts along the dimer. The residues I36, Y38, P39, L140, Y165, I167 and V170 also mediate weak contacts between protomer domains, thus conferring stability to the dimer. Furthermore, non-covalent interactions involve the core I36 with the main chain of the β-hairpin residues S166, I167, R168 and G169. R168 is also involved in H bonds with the backbone carbonyls of the core P162 and K172. A considerable number of amino acids in this loop interact with the opposing domain. This amino acid sequence is specific for NAA60, and no other NAT exhibits this observed homodimerization propensity [32, 93]. X-ray data were also corroborated by size-exclusion chromatography assessing peptide substrate-dependent monomer–dimer transition in vitro [89]. However, no studies have yet described the aggregation propensity of NAA60 in vivo, and oligomerization of the protein in physiological and pathological cell environments warrants investigation.

### NAA60 is a Golgi acetyltransferase

Although known as the power factory of PTMs, the Golgi apparatus is still not described in the literature as harbouring any acetyltransferase enzymes. However, exploratory bioinformatics investigations reported that NAA60 contains putative transmembrane domains (TMDs) in its C-terminal region [40]. Indeed, after deleting these domains, NAA60 dislodgement and Golgi apparatus fragmentation and dispersion in the cytoplasm are observed, suggesting that NAA60 is anchored on the Golgi membrane stacks [40, 94]. The subcellular distribution of NAA60 was assayed in vitro in cells expressing all known NATs with a C-terminal V5 tag [40]. Differently from other known NAA enzymes which are characterized by cytoplasmic and nuclear localizations, NAA60 has a typical organelle localization pattern. Indeed, co-localization analyses revealed that V5-tagged NAA60 was co-distributed with the *cis*-Golgi protein GM130 and the *cis*/medial-Golgi marker giantin. Additional proof for NAA60 occupancy of the Golgi apparatus came from the finding that organelles are sensitive to the Golgi-destroying drug brefeldin A: upon incubation of engineered cells with this compound, immunofluorescence experiments against NAA60 showed that the protein was disseminated in vesicles throughout the cell. The fragmented membranous structures were co-localized with fluorescent antibodies for endosomes, lysosomes, peroxisomes and secretory vesicles [86]. Specific regions unique to NAA60 owing to its peculiar organelle localization have been identified. By sequence alignment [31] and by comparing NAA60 with NAA50 crystal structures [93], some regions warranting further investigation were found, as described in more detail in the previous sections. In particular, the end region (residues 216–221) contains a putative endosome/lysosome-targeting peptide sequence (QAHSLL). Furthermore, the C-terminal arm harbours two putative TMDs in segments 193–213 and 217–236. Two putative S-palmitoylation sites on C207 and C222 were also identified, and truncation and deletion mutants were characterized. Among these, a NAA60 variant missing the last 58 amino acids lost membrane localization, showing that the C-terminal region is necessary for the proper targeting of NAA60 at the subcellular level. Upon removal of the last 26 amino acids, the same localization as the full-length version was not observed. A truncated NAA60 variant at the NH_3_ tail also showed impaired patterns of localization, but retained membrane targeting. Deletion of Sects. 78–87 and 216–221, as well as the point mutation of L220A and L221A, did not affect the subcellular localization of the protein. Selective membrane permeabilization experiments showed that NAA60 faces the cytosol side.

Given the unusual Golgi localization of NAA60, the subcellular distribution of its targets has been also analysed. Proteome analyses of soluble and insoluble proteins of siRNA-treated cells identified around 1700 unique N-terminal substrates, of which more than 200 are transmembrane proteins and more than 20 have been proposed as potential NAA60 substrates [40]. The candidate NAA60 substrates are expressed in manifold membrane compartments, including cytoplasmic membrane, endoplasmic reticulum, mitochondria, Golgi apparatus and vesicles. In vitro acetylation assays using recombinant NAA60 and synthetic N-terminal peptides supported that the Met-starting N-termini were direct substrates of NAA60.

NAA60 knockdown induces Golgi ribbon fragmentation. Given the finding that NAA60 acetylates transmembrane proteins [90], it is possible that N-terminal acetylation of this category of targets is directly or indirectly involved in the structural organization of Golgi ribbons. Alternatively, Golgi disruption might be linked to cytoskeleton-related effects, since microtubules and actin play key roles in maintaining organelle integrity and distribution during cell life [95, 96]. Golgi fragmentation occurs in a regulated manner during both mitosis and apoptosis. However, in mitosis the mammalian Golgi apparatus continuously undergoes structural reorganization. Rebuilding of the Golgi ribbon is also linked to a G2/M restriction checkpoint [97]. Given the Golgi phenotype in *NAA60*^−/−^ human cells and lagging chromosome formation found in Drosophila, it has been suggested that the protein may be involved in chromosome segregation during anaphase in mitosis [31].

### The role of NAA60 in cell life

First knock-down experiments in Drosophila opened the survey of the role of NAA60 in cell life. Similarly, to NAA50 silencing [98], cells showed unpaired chromosomal segregation during anaphase. However, unlike *NAA50*-depleted cells, which exhibit abnormal metaphases, the knocked-down cells showed the expected metaphase, where all chromosomes are perfectly aligned and no mitotic arrest occurred. However, during anaphase, consistent segregation errors relating to centrosome/mitotic spindle defects were observed in NAA60-depleted cells. NAA60-dependent acetylation of one or more substrates is therefore thought to be required for chromosome segregation in vivo [31]. To investigate whether NAA60 exerts its activity on histones, recombinant and isolated engineered mammalian NAA60 were treated with commercial histones or extracted endogenous nucleosome members in the presence of Ac-CoA. Whilst the recombinant NAA60 indiscriminately acetylated all histone species, the purified endogenous mammalian NAA60 preferentially acted on histone H4. Specifically, the mammalian protein acetylates lysines 20, 79 and 91 of histone H4. The fractionated Golgi incubated with commercial histones or nucleosome histones in the presence of Ac-CoA showed HAT activity, confirming that NAA60 exerts its activity in the Golgi apparatus. Many known HATs require autoacetylation for HAT activity. In line with this observation, NAA60 is autoacetylated on lysines 79, 105 and 156 [86].

In vitro, the recombinant NAA60 targeted numerous histones and a high number of amino acids, including lysines 5, 12, 20, 31, 77, 79 and 91 on H4, lysines 56 and 122 on H3, lysines 34 and 46 on H2B and lysine 5 on H2A. Differently, the mammalian NAA60 almost exclusively acetylated lysines 12, 20, 79 and 91 on histone H4. [86]. The ability to acetylate the buried H4 lysines 79 and 91 is intriguing. Most histone acetylations, whether catalysed by A-HATs or B-HATs, are directed at external histone tails, and not in the globular concealed domain where K79 and K91 are located. However, modifications on histone globular domains, due to their involvement in histone–histone interactions and octamer oligomerization [99, 100], are probably more relevant or nucleosome organization despite the histone tails. NAA60 can thus be considered an “epi-enzyme” for its involvement in chromatin assembly, as HAT1 [10]. As regards a hypothesized crosstalk between HAT1 and NAA60, upon cell synchronization treatment in HeLa cells the expression profiles of HAT1 and NAA60 were different during cell cycle progression, with a decreased expression of HAT1 and a counteracting higher expression of NAA60. In addition, HAT1 expression peaked at G1/S phase, whereas NAA60 expression increased at the S/G2/M checkpoint. Based on these findings and on the substrate specificity of both enzymes, it could be hypothesized that the two enzymes exert overlapping but non-redundant activities in cell life [86].

### Role of NAA60 in cell proliferation

Compromised euchromatin and heterochromatin organization and subsequent unpaired genome expression inevitably alter cell growth and general homeostasis [101]. Flow cytometry analysis revealed that NAA60 is involved in regulating cell cycle progression [86]. Mistakes in DNA synthesis coupled with the disruption of nucleosome assembly are partially associated with high exposure to acute DNA damage [101, 102]. For example, NAA60-depleted human cells exposed to DNA-damaging treatments with hydroxyurea, ultraviolet irradiation and camptothecin showed NAA60 loss of function leading to significantly decreased cell viability. Co-knockdown of both HAT1 and NAA60 markedly reduced cell number, since NAA60 depletion is highly sensitized to DNA damage [86]. The effects observed in *NAA60*^*−/−*^ cells could be due to the inability to adequately organize the whole genome or might be a consequence of apoptosis triggered by *NAA60* knockdown. The effect of NAA60 on apoptosis has also been investigated. HAT1 and *NAA60* siRNA human cells were chemically blocked at the S phase of the cell cycle. Cytofluorimetry analysis revealed that HAT1/NAA60 knockdown led to an accumulation of cells undergoing apoptosis. Western blotting showed that NAA60-depleted cells highly expressed the apoptotic marker p53 and phosphorylated H2AX, corroborating NAA60-mediated activation of the apoptotic pathway [86]. Collectively, these findings support the hypothesis that NAA60 is critically involved in normal cell functioning and is directly dependent on proper chromatin structural organization.

### NAA60 in disease

NAA60 is a newly characterized acetyltransferase and our understanding of its role in pathogenic pathways has not yet been fully explored. As annotated on the GeneCards database, diseases associated with NAA60 include Scheuermann’s disease and syndromic microphthalmia-1, although there is currently no direct evidence based on biomedical research.

A recent study reported the role of the NAA60 protein in influenza A virus (IAV) infection of lung cells [103]. NAA60 was found to play a proviral role during IAV infection by disrupting interferon IFNα signalling [104]. This finding was obtained by NAA60 overexpression and knockdown experiments. In NAA60-depleted cells infected with IAV particles, the release of viral progeny in cells was reduced by about 50%; the opposite result was observed when NAA60 was overexpressed. When NAA60 was downregulated, mRNA levels of IFNα were upregulated, whilst no changes were found in IFNβ and IFNγ expression. These data indicate that NAA60 hampers the expression of IFNα upon virus infection. Since IFNα, but not IFNβ or IFNγ, signalling is induced at the first stage of infection, NAA60 is thought to be involved in early anti-IAV signalling events. Activation of the INFα pathway modulated by NAA60 has various feedback effects, including phosphorylation and subsequent activation of the transcription factor STAT1. In contrast, depletion of NAA60 enhances levels of phosphorylated STAT1. Upon activation, STAT1 translocates to the nucleus and drives the expression of IFN-stimulated genes (ISGs) such as IFN-induced transmembrane (*IFITM*) protein 1, 2 and 3, *ISG15*, cholesterol 25-hydroxylase (*CH25H*), tripartite motif protein 22 (*TRIM22*), myxovirus resistance protein 1 (*MX1*), viperin and mitochondrial antiviral signalling protein (*MAVS*). Similar results were observed in *NAA60*-deficient cells treated with purified IFNα. Taken together, these findings corroborate the hypothesis that NAA60, and N-terminal acetylation in general, has a pivotal role during IAV infection and virus replication [104]. The observed behaviour of NAA60 during inflammation cascades mediated by IFNs is also shared by other enzymes modifying lysines upon viral infections [105]. The activation/repression of IFN-dependent pathways involving this category of enzymes might result in the stimulation of Toll-like receptor cascade signalling, whose key role is recognized in the pathophysiology of inflammation as well as in cancer progression [106].

These recent shreds of evidence are consistent with the well-known proviral role of other acetyltransferases, such as p300/CBP [107], NAA20/25 [108], Gcn5 and PCAF [109], and are complemented by the known antiviral action of several deacetylases, including sirtuins [110], HDAC1 [111], HDAC2 [112], HDAC4 [113], HDAC6 [114] and HDAC11 [115], during IAV infection. Gcn5 and PCAF acetyltransferase activities are also associated with suppressed expression of type I IFN and ISG targets such as viperin, STAT1 and MX1 [109]. Similarly, the p300/CBP complex is a key regulator of the expression profiles of numerous host proteins acting at various stages of the IAV life cycle [116]. NAA20 / 25, GCN5 and PCAF regulate IAV polymerase and host quenching activities by acetylating viral proteins [108, 117, 118], whilst HDAC6 [118] and HDAC11 [115] enhance IAV-induced type I IFN production. Additionally, HDAC 1, 2, 4, and 11 increase STAT1 phosphorylation as well as expression of ISGs, such as IFITM3, viperin and ISG15, upon viral infection [111–113, 115]. Since NAA60 is anchored to the Golgi membrane and given that viperin [119] and CH25H [120] are membrane-associated proteins and localize to the endoplasmic reticulum and Golgi apparatus, the proviral action of NAA60 might be quite easily understood. Viperin [121] and CH25H [122] actions have relevant roles in virus replication in cells for their enrolment in lipid synthesis and organization in membrane. Thus, as hypothesized by the first studies on cells [104], under virus stimulation NAA60 might influence the function of viperin and CH25H upon their N-terminal acetylation in the Golgi complex since they are upregulated in NAA60-depleted cells among all the ISGs. At the same time, NAA60 behaviour might also have direct or indirect drawbacks on the function of the Golgi-localized IAV proteins HA and NA.

## Conclusions

N-terminal acetylation is the most frequent PTM occurring in a variety of protein species. It drives the modulation of a wide range of biochemical and biological events in cells, under both physiological and pathologically compromised conditions. Over the years, several acetylating enzymes have been discovered and extensively characterized across the phylogeny, along with their direct targets and cross-talking signalling pathways. NAA60 is one of the newly discovered N-terminal acetyltransferases that is found to have biochemical properties shared by both B-HATs and NATs. Unlike all other known HATs and NATs, NAA60 displays novel and highly unusual properties. First, it seems to be able to transfer acyl groups from Ac-CoA to newly synthesized histone lysines (both those more exposed on N-tails and those buried in the H4 globular domain) as well as to peptide substrates starting with M–K/M-A/M-V/M-M residues. It thus exhibits dual enzymatic HAT/KAT and NAT behaviour. Surprisingly, this new acetyltransferase is localized in the Golgi membrane stacks facing the cytosol; its enzymatic action is exploited at its C-terminal domain. Structural investigations found another fascinating feature associated with its aggregation propensity: when NAA60 is active it is in monomeric form, where the catalytic site is free to transfer acyl groups to peptide substrates. Otherwise, it can exist as a dimer upon peptide binding, thus avoiding another Ac-CoA molecule to access on the catalytic residues. Although a detailed investigation on NAA60 biological roles ruled in the cell is not deeply carried out, it is currently reported to orchestrate free H4 assembly in the final nucleosome octamer, thus regulating chromatin organization and cell cycle progression.

Gaining a greater insight into NAA60 and this class of epi-enzymes, the knowledge about acetyltransferases represents a new challenge in the biochemistry of the acetylome and in the field of personalized medicine. Particularly, in the scenario of severe diseases, such as cancer, NAA60 is a fascinating Golgi bi-functional acetyltransferase worthy of further characterization both in vitro and in vivo given its intriguing epigenetic role and its promising biomedical application in the design of new selective molecules directed against this novel epigenetic target. Understanding the molecular mechanisms underlying the unusual dual behaviour as HAT and NAT may allow us to rule out the activation and the progression of peculiar biological events directly modulating the gene expression at the epigenetic level, especially in diseases and disorders strictly related to environmental alterations.

## Data Availability

Not applicable.

## Abbreviations

PTMs: Post-translational modifications
HAT: Histone acetyltransferase
KATs: Lysine (k) acetyltransferases
Naa: N-alpha-acetyltransferase
Nat: N-acetyltransferase
NAT: N-acetyltransferase
KDACs: Lysine (K) deacetylases
HDACs: Histone deacetylases
SIRT: Sirtuin
Ac-CoA: Acetyl coenzyme A
NAD +: Nicotinamide adenine dinucleotide
GCN5: General control nonderepressible 5
GNAT: General control nonderepressible 5-related N-acetyltransferase
MYST: Histone acetyltransferase monocytic leukaemia
CPB: CREB binding protein
MOF: Males absent on the first
NuA4: Multisubunit nucleosomal acetyltransferase of H4
Tip60: Tat interactive protein 60 kDa
DNA-PKcs: DNA-dependent protein kinase catalytic subunit
MSL: Multisubunit male-specific lethal
NSL: Human non-specific lethal
Bax: Bcl-2-associated X
Puma: P53 upregulated modulator of apoptosis
EGFR: Epidermal growth factor receptor
ATG7: Autophagy-related 7
CENP-A: Centromere protein A
M18BP1: Mis18-binding protein 1
INK4a: INhibitors of CDK4
ARF: Alternative reading frame
Cdc6: Cell division cycle 6
Ezh2: Enhancer of zeste homolog 2
E2F: Elongation factor 2
Melk: Maternal embryonic leucine zipper kinase
MAPK: Mitogen-activated protein kinase
PGC-1α: Peroxisome proliferator-activated receptor-γ coactivator-1a
STAT: Signal transducer and activator of transcription
BRCA1: BReast CAncer gene 1
FOXO 1/3a/4: Forkhead box protein O
SMA: Spore membrane assembly
MAD: Mothers against decapentaplegic
HIF-1α: Hypoxia-inducible factor 1 subunit alpha
RUNX: Runt-related transcription factor
Mdm2: Mouse double minute 2 homolog
mRNA: Messenger RNA
CEP170: Centrosomal protein 170
NSCLC: Non-small-cell lung cancer
Nrf2: Nuclear factor erythroid 2-related factor 2
CDK: Cyclin-dependent kinase
Erk1/2: Extracellular signal-regulated kinase1/2
TGFβ: Transforming growth factor beta
Wnt: Wingless-related integration site
Dkk1: Dickkopf WNT signalling pathway inhibitor 1
BRD4: Bromodomain-containing 4
PBX3: Pre-B cell leukaemia transcription factor 3
SENP6: SUMO-specific peptidase 6
MEIS1: Meis homeobox 1
HCCs: Hepatocellular carcinomas
PCNA: Proliferating cell nuclear antigen
AIB1: Amplified in breast cancer 1
Egr-1: Early growth response 1
PIX: PAK (p21-activated kinase) Interacting eXchange factor
Cdc42: Cell division control protein 42
Rac1: Ras-related C3 botulinum toxin substrate 1
Hippo: Salvador–Warts–Hippo (SWH) pathway
YAP: Yes-associated protein
PP2AC: Protein phosphatase 2 catalytic subunit alpha
Grb2: Growth factor receptor-bound protein 2
IAV: Influenza A virus
INF: Interferon
ISGs: IFN-stimulated genes
IFITM: IFN-induced transmembrane
CH25H: Cholesterol 25-hydroxylase
TRIM22: Tripartite motif protein 22
MX1: Myxovirus resistance protein 1
MAVS: Mitochondrial antiviral signalling protein
PCAF: P300/CBP-associated factor
TSC2: Tuberous sclerosis complex 2
mTOR: Mechanistic target of rapamycin
K: Lysine
M: Methionine
S: Serine
A: Alanine
T: Threonine
G: Glycine
V: Valine
D: Aspartate
E: Glutamate
N: Asparagine
L: Leucine
I: Isoleucine
F: Phenylalanine
W: Tryptophan
